# Airborne Gold Nanoparticle Detection Using Photoluminescence Excited with a Continuous Wave Laser

**DOI:** 10.1177/00037028211042021

**Published:** 2021-09-03

**Authors:** Per Samuelsson, Markus Snellman, Martin H. Magnusson, Knut Deppert, Marcus Aldén, Zhongshan Li

**Affiliations:** 1 Division of Combustion Physics, Lund University, Lund, Sweden; 2 Division of Solid State Physics and NanoLund, Lund University, Lund, Sweden

**Keywords:** Gold nanoparticles, photoluminescence, laser excitation, aerosols, in situ imaging

## Abstract

We report the observation of photoluminescence emission from airborne gold, silver, and copper nanoparticles. A continuous wave 532 nm laser was employed for excitation. Photoluminescence from gold nanoparticles carried in a nitrogen gas flow was both spectrally resolved and directly imaged in situ using an intensified charge-coupled device camera. The simultaneously detected Raman signal from the nitrogen molecules enables quantitative estimation of the photoluminescence quantum yield of the gold nanoparticles. Photoluminescence from metal nanoparticles carried in a gas flow provides a potential tool for operando imaging of plasmonic metal nanoparticles in aerosol reactions.

## Graphical abstract

**Graphical Abstract:**
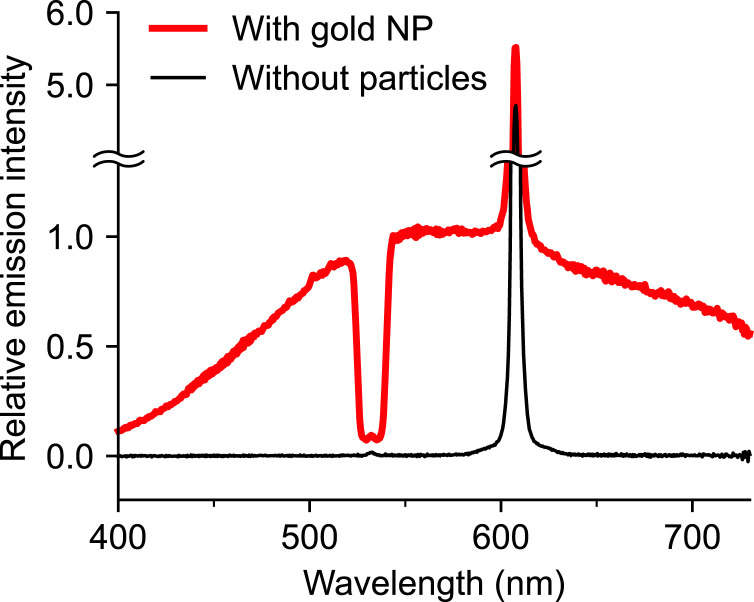

## Introduction

Gold nanoparticles have found use in numerous applications both in research and industry, including optics,^1^ sensing,^2^ catalysis, and as seed crystals for surface-^3^ and aerosol-supported nanowire growth,^4–6^ much because of its non-reactive properties. Compared to surface growth, gold-seeded nanowires grown by aerosol-based methods have demonstrated a superior growth rate.^5^ The transition from surface to gas phase growth, however, means that new diagnostic tools are required, and an inherently continuous growth method demands on-line in situ diagnostics for efficient control. Whereas photoluminescence is a routine tool for assessing semiconductor crystallinity, it has not been commonly applied to metals due to the low photoluminescence yield in bulk metal, resulting from fast nonradiative relaxation processes.^7^ This limitation can be overcome thanks to plasmonic effects in metal nanoparticles, i.e., the collective oscillation of the conduction electrons, driven by an external field.

Photoluminescence in metals was first reported by Mooradian^8^ as a broadband emission from solid gold and copper surfaces following continuous wave (CW) laser irradiation and was explained as excitation of the *d*-band electrons to the *sp* conduction band, above the Fermi energy, followed by relaxation, and radiative recombination of electrons and holes. The quantum efficiency was reported to be in the order of 10^−10^.

A systematic study of both smooth and rough surfaces was carried out by Boyd et al.,^9^ and a theoretical explanation for the bulk effect was given, later expanded by Apell et al.^10^ The increased response from higher aspect ratio features on rough surfaces was attributed to a local-field enhancement effect, analogous to the enhancement process in surface-enhanced Raman spectroscopy (SERS). Later, this theory was used to explain a red shift and increasingly enhanced photoluminescence intensity with increasing aspect ratio of gold nanorods.^11^

Another explanation is given in a study by Dulkeith et al.,^7^ where the authors argued that the final step in plasmonic nanoparticles is instead nonradiative recombination of the electron–hole pair, producing a particle plasmon, which is then emitted as a photon, resulting in plasmon-enhanced photoluminescence emission. A quantum yield of 10^−6^ was found for gold nanoparticles. The spectral shape of the photoluminescence emission was reported to closely follow the theoretical Mie extinction spectrum of the particles investigated.

In this work, we investigate the photoluminescence emission from airborne metal nanoparticles excited with a 532 nm CW laser, with focus mainly on gold but also copper and silver nanoparticles generated in the same spark discharge system. The Raman signal from the nitrogen carrier gas is detected simultaneously and, being of a comparable intensity and experiencing identical optical paths as the photoluminescence signal, it acts as a convenient scale for the estimation of the photoluminescence quantum yield.

## Experimental

**Figure 1.**
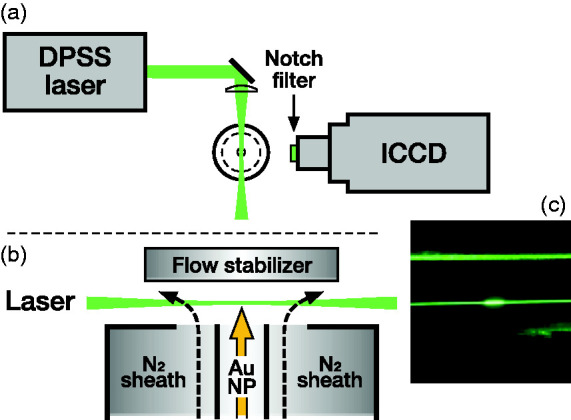
(a) Schematic of the experimental setup for imaging of airborne metal nanoparticles, as seen from above. For spectroscopic measurements, the ICCD camera was replaced with a spectrometer, with the slit aligned along the excitation beam. (b) Magnified cross-section from the camera’s point of view. Optical measurements on gold nanoparticles are carried out in open air, protected by a nitrogen sheath flow. (c) Photograph depicting elastic Mie scattering from airborne gold nanoparticles (central bright spot), and molecular Rayleigh scattering from nitrogen along the excitation laser beam. The green excitation light scattered off the flow stabilizer is visible above the laser beam.

Metal nanoparticles were generated in a spark discharge aerosol generator (SDG).^12^ The discharge was driven by a high-voltage (HV) supply that continuously charges a capacitor bank. Whenever the voltage over the spark gap exceeds the breakdown threshold of the carrier gas between the electrodes, the capacitor bank is discharged, and a conductive channel is formed across the spark, heating up and ablating metal vapor from the electrodes. The metal vapor is subsequently quenched by the carrier gas, condensing to nanoparticles, which are transported using the carrier gas to the optical setup, as shown in Fig. 1. The particles coming out of the aerosol generator are generally not individual spherical particles but agglomerates.^13^ The total capacitance of the capacitor bank was 20 nF. The high voltage supply (Model 15KV-150 J, Technix, France) was operated at 12 kV voltage setpoint and 10 mA charging current, which determines the spark repetition rate. The breakdown voltage was typically around 7 kV, depending on the spark gap and geometry. The electrodes were 3 mm diameter cylindrical gold (Au), silver (Ag), and copper (Cu) rods (Goodfellow, UK) at 2 to 3 mm gap between the electrodes. One electrode was electrically connected to the HV output of the capacitor bank, the other one was grounded. The separation between the two electrodes was adjustable. The carrier gas was 1.5 L min^−1^ pure nitrogen (99.9999% N_2_) at ambient pressure, with a flow speed of about 2 m s^−1^ inside the tubes. The size distribution of gold nanoparticles was measured in a separate experiment, using a scanning mobility particle sizer (SMPS), consisting of an Ni-63 bipolar charger, electrostatic classifier (EC, Model 3082, TSI), differential mobility analyzer (DMA, Model 3081, TSI), and a condensation particle counter (CPC, Model 3775, TSI). In order to avoid saturation of the CPC, the particle flow was diluted by a factor 6.3, downstream of the SDG. No dilution was used during optical measurements.

In situ optical measurements were performed in an open-air vertical flow geometry (Fig. 1). A nitrogen sheath flow and a flow stabilizer were employed to minimize interference from aerosol dust and oxygen in ambient air, with an approximate sheath/core flow speed ratio of 2. A narrowband 532 nm CW neodymium-doped yttrium orthovanadate (Nd:YVO_4_) diode-pumped solid state laser (Sprout 15 W, Lighthouse Photonics) was used as excitation source. The laser beam was loosely focused about 5 mm above the exit of the particle flow tube using an *f* = 500 mm spherical lens, resulting in an estimated beam waist diameter of about 90 µm, and a maximum power density of 2.4 × 10^5 ^W cm^−2^ at 15 W laser output power. This is insufficient to optically trap particles given drag from the carrier gas flow, but likely results in particle melting and coalescence, which will be discussed in a later section. Shown in Fig. 1c is a photograph of the Mie scattering from the nanoparticle flow, and Rayleigh scattering from the ambient gas, illuminated by the 532 nm laser beam.

A notch filter with a specified optical density > 6.0 at 533 nm (NF533-17, Thorlabs) was employed to block the strong elastic Mie scattering from the nanoparticles and Rayleigh scattering from the carrier gas (visible in Fig. 1c) at the excitation laser wavelength. The remaining photoluminescence emitted by the metal particles and the spontaneous Raman scattering from the nitrogen molecules was detected perpendicularly to the laser beam and imaged 1:1 onto the horizontally oriented entrance slit of a grating spectrometer using an *f* = 100 mm fused silica lens. An aperture was employed to reduce the collection angle to approximately 7° from the optical axis. The photoluminescence emission was spectrally resolved using an *f* = 150 mm, *f*/4 spectrometer (SP150, Princeton Instruments), equipped with a 300 lines mm^−1^ grating and an intensified charge-coupled device (ICCD) camera (PIMAX4, Princeton Instruments). The slit width was 140 µm; spectral resolution was 3.6 nm. The instrument response of the spectrometer was determined using a tungsten-halogen calibration lamp (IES1000, LabSphere). In addition to spectrally resolved measurements, direct images were collected at different excitation laser intensities, using the same ICCD camera used for spectroscopy, through replacing the spectrometer with an *f* = 105 mm imaging lens (UV Nikkor), stopped down to *f*/8 and mounted on a 42 mm extension tube. The resulting spatial resolution was about 30 µm per pixel. No visible image degradation was observed due to chromatic aberrations. Direct imaging was limited by the sensor response to emission wavelengths longer than approximately 350–400 nm. The polarization direction of the linearly polarized excitation laser beam was controlled using a half-wave plate, and measurements were performed both with the laser polarized normal to the imaging plane (horizontally polarized) and parallel to the imaging plane (vertically polarized) in order to determine the polarizability properties of the photoluminescence emission and to scale its intensity by the well-known spontaneous Raman scattering from nitrogen molecules.

## Results and Discussion

**Figure 2.**
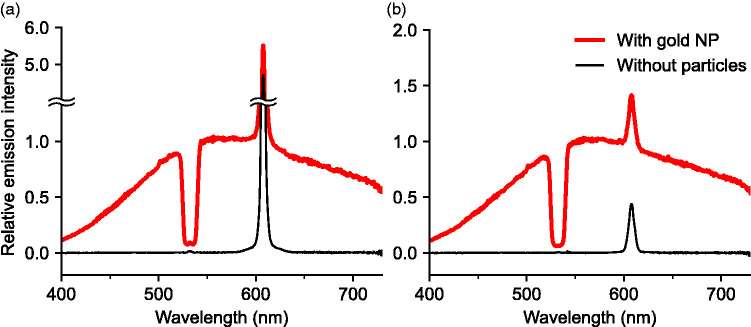
Emission from gold nanoparticles, excited with (a) vertically and (b) horizontally polarized laser at 15 W. The red spectral curves constitute the collected emission with gold nanoparticles in the nitrogen flow, the black curves were collected with nitrogen only. The sharp peak near 607 nm is the Stokes-shifted nitrogen Raman signal resonant at 2331 cm^−1^, and the dip centered on 533 nm was caused by the notch filter. The small tip in the middle of the spectral notch of the interference filter was due to the strong elastic Mie scattering leaking through the filter. The total integration time was 10 s for each spectrum.

Spectrally resolved emission excited with the loosely focused 532 nm CW laser beam was collected and led to the spectrometer. Shown in Fig. 2 are results with gold nanoparticles and 15 W excitation laser power. By switching on and off the SDG, the existence of gold nanoparticles in the detection volume can be readily controlled. With no particles in the flow, only the nitrogen Raman signal was observed. The residual Raman signal observed using horizontal polarization is due to finite depolarization of nitrogen and the residual vertically polarized component of the laser beam.

With the SDG on, the photoluminescence from the gold nanoparticles was recorded as a broad spectral distribution extending over the entire visible wavelength range, as shown by the red spectral curves, and to both higher and lower energy compared to the excitation wavelength. The photoluminescence emission is independent of laser polarization, unlike elastic Mie and Rayleigh scattering which are highly polarized. It may be noted that the grating has a slight polarization preference, but this is much smaller than the observed ratio between the nitrogen Raman signals for vertically and horizontally polarized excitation laser, with a value of approximately 11, as shown in Fig. 2. The intensity of the broadband emission is comparable to the nitrogen Raman signal, which is in the order of 10^4^ times weaker than the Mie scattering from the particles.

If the number density and absorption cross-section of the particles are known, we can calculate the efficiency of the photoluminescence emission using the nitrogen Raman signal as reference. To calculate the photoluminescence efficiency, we measured the relative intensities of the photoluminescence emission and the Raman scattering. With a known particle size distribution, the photoluminescence quantum yield is then given by

(1) $$\Phi_{\text{PL}}=4\pi (\frac{I_{\text{PL}}}{I_{\text{R}}})\frac{n_{\text{gas}}(\frac{\text{d}\sigma R}{\text{d}\Omega })}{\int_{0}^{\infty }n_{\text{part}}(a)\sigma_{\text{abs}}(a)\text{d}a}$$

where (*I*_PL_/*I*_R_) is the ratio between the measured photoluminescence emission and Raman scattering intensities, *n*_gas_ is the nitrogen molecule number density, (dσ_R_/dΩ) = 0.46 × 10^−30^ cm^2^ sr^−1^ molecule^−1^ is the differential Raman cross-section of nitrogen at the excitation wavelength,^14^ *n*_part_(*a*) is a function describing the number density of nanoparticles with radius *a*, such that $\int_{0}^{\infty }n_{\text{part}}(a)\text{d}a$ is the total number density, and σ_abs_(*a*) is the Mie absorption cross-section,^15^ assuming spherical particles. The emission intensities *I*_PL_ and *I*_R_ of photoluminescence and Raman scattering were found by integrating the respective curves in Fig. 2a, linearly interpolating over the notch in the case of *I*_PL_.

**Figure 3.**
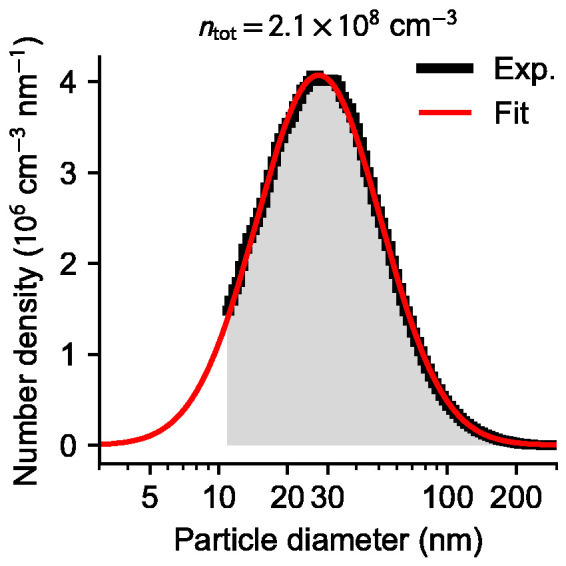
Mobility size distribution of gold nanoparticles (black curve) determined using an SMPS system, and a log-normal fit (red curve). The fit was used in calculation of particle absorption cross-sections, assuming spherical particles.

The expression for the photoluminescence quantum yield in Eq. 1 requires accurate knowledge of the particle size distribution. The gold nanoparticle size distribution was estimated using an SMPS and a typical result is shown in Fig. 3. The SMPS determines the mobility diameter of the particles, which is identical to their geometric diameter for spherical particles only. The particles generated in our spark discharge generation system are most likely aggregates, and consequently the particle mobility size distribution shown in Fig. 3, and by extension the calculated photoluminescence quantum yield, which assumes spherical particles, constitute the best estimates based on available information.

The photoluminescence quantum yield of the gold particles was estimated from spectra collected with the laser vertically polarized. Using the relative intensity between the photoluminescence and the Raman signals shown in Fig. 2a, (*I*_PL_/*I*_R_) ≈ 20, and a log-normal fit to the size distribution shown in Fig. 3, the gold nanoparticle photoluminescence quantum yield was estimated using Eq. 1 to be in the order of 10^−7^, which is within the expected range for gold and the same order of magnitude as reported for single particles.^16^ A major source of uncertainty is the size distribution of the particles, as well as their shape, which could influence the calculated absorption cross-section.

**Figure 4.**
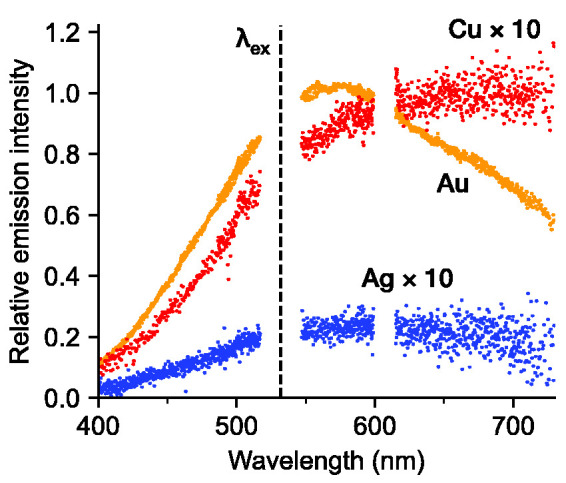
Emission spectra from Au, Cu, and Ag nanoparticles, with the laser horizontally polarized. The gold and copper spectra were collected under the same experimental conditions, while the silver spectrum was collected with a higher discharge energy to increase the silver nanoparticle concentration. The excitation laser wavelength was λ_ex_ = 532 nm.

Shown in Fig. 4 are photoluminescence emission spectra from gold, copper, and silver nanoparticles detected with the same optical system and with the laser beam horizontally polarized. The gold, copper, and silver particles were introduced through spark ablation of the corresponding electrode material. Sample points in the vicinity of the excitation laser wavelength, 532 nm, and the Raman signal near 607 nm, have been removed to better elucidate the photoluminescence signals. The intensity of the copper and silver spectra has been enhanced by a factor 10. Out of the three metals measured, the gold particles resulted in the strongest photoluminescence signal, which could be due to several favorable factors including the location of the plasmon resonance, band structure, and resistance to oxidation. The fact that the excitation wavelength is closer to the inter-band energy in gold compared to silver or copper may explain the stronger response. Additionally, under equivalent conditions, the ablated gold mass can be expected to be a factor three times that of silver or copper.^12^ During the gold and copper measurements, the SDG was operated at 2 mm spark gap, whereas a 3 mm spark gap was used for silver in order to get sufficient signal, resulting in a correspondingly higher discharge voltage and electric energy deposition. The intensity of the Au nanoparticle photoluminescence is a factor 20 times stronger than the Raman signal within the detected wavelength range, whereas both copper and silver are an order of magnitude weaker than gold. In order to be comparable, however, the photoluminescence intensity should be normalized by the respective particle volume fraction, which is unknown for silver and copper particles.

**Figure 5.**
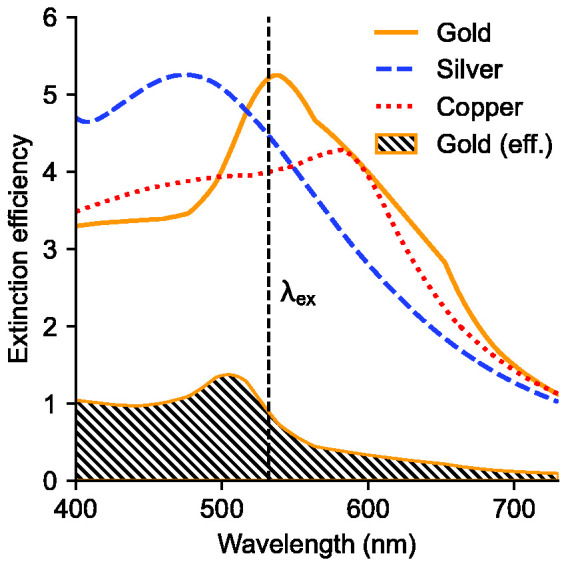
Calculated Mie extinction efficiencies of 150 nm diameter gold, silver, and copper spheres. The hatched curve represents the effective Mie extinction efficiency, based on the gold nanoparticle mobility size distribution shown in Fig. 3 (red curve), which peaks at 30 nm diameter, assuming spherical particles.

The spectral curves exhibit broad maxima, peaking around 550–600 nm for gold, whereas copper and silver both appear to peak at longer wavelengths. In the case of gold, the inter-band absorption edge is about 2.2 eV, corresponding to about 560 nm, and for copper 2.0 eV or 620 nm.^8^ Dulkeith et al.^7^ reported that the spectral shape of the photoluminescence emission from spherical particles agrees with the theoretical Mie extinction efficiency for the particles studied. Our measurements instead indicate a red shift of the gold photoluminescence emission peak with respect to the calculated Mie extinction spectrum, as illustrated in Fig. 5, which shows the estimated effective Mie extinction efficiency (hatched) given the measured mobility size distribution of gold, as shown in Fig. 3. Also shown in Fig. 5 are the calculated Mie extinction efficiencies of gold, silver, and copper spheres of 150 nm diameter, which more closely matches the observed spectra. Refractive index data used in the Mie calculations was obtained from Palik et al.^17^ (gold, silver) and Johnson and Christy^18^ (copper). The governing mechanism of the photoluminescence emission from metal nanoparticles is still to be discovered in future research.

**Figure 6.**
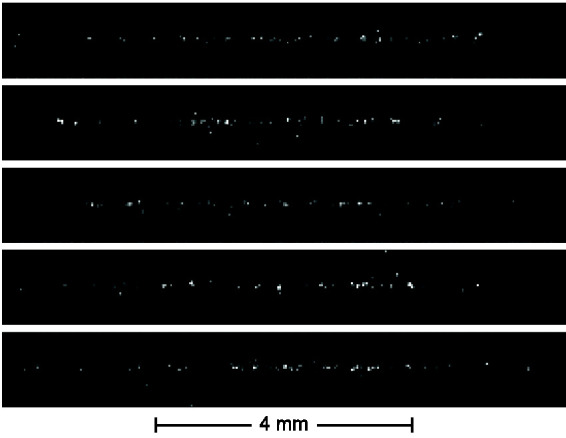
Sequence of single-shot photoluminescence images of gold nanoparticles in the flow, at 100 μs integration time.

Direct images of gold nanoparticles in the carrier gas flow were recorded using an ICCD camera with a notch filter installed to block the elastic scattering. The laser beam was horizontally polarized. Shown in Fig. 6 is a sequence of single-shot direct images recorded at 15 W laser power and 100 μs integration time. The images exhibit grainy structures that we tentatively attribute to individual large particles or agglomerates. Correspondingly, spectra of outstanding strong individual particles are seen occasionally in the spectra, which were removed prior to averaging.

**Figure 7.**
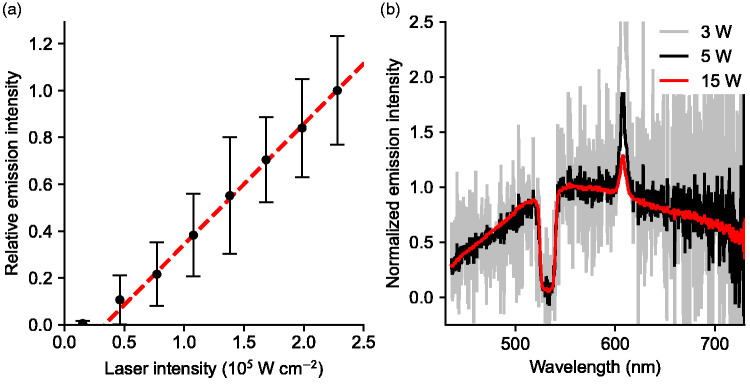
(a) Au nanoparticle photoluminescence intensity as a function of the incident laser intensity. The error bars correspond to one standard deviation, where each sample was taken as the mean intensity of each frame, out of 1000 frames collected at each power setting. Less than 10 out of 1000 frames have been omitted at each power, due to occasional presence of larger particles. (b) Spectrally resolved Au photoluminescence intensity for different laser intensities.

Shown in Fig. 7a is the photoluminescence intensity emitted from gold nanoparticles, recorded at different excitation laser powers. Under the pertaining experimental conditions, there is a threshold below which no photoluminescence was observed, which occurred near 0.16 × 10^5 ^W cm^−2^ for gold. Above 0.8 × 10^5 ^W cm^−2^, the emitted intensity is nearly linear in the excitation laser power up to 2.4 × 10^5 ^W cm^−2^, which was the maximum power density used. We speculate that the threshold may be due to removal of particle surface oxidation or reshaping due to melting. Shown in Fig. 7b are spectra of the gold nanoparticle photoluminescence emission, excited with different excitation laser powers. The results indicate that the spectral shape of the Au nanoparticle photoluminescence emission is independent of excitation power, and the integrated intensity close to linear in incident laser intensity, except for a threshold at the lowest laser powers used. While this evidence suggests that photoluminescence may be responsible for the observed broadband signal, other possible explanations, besides photoluminescence, will be further addressed in the following.

Elastic scattering in the form of molecular Rayleigh scattering from the carrier gas and Mie scattering from nanoparticles are efficiently suppressed by the narrow notch filter centered on 533 nm. The spectral bandwidth of the laser is negligible. Elastic Mie scattering from the gold particles was observed to be approximately 20 times more intense than the Rayleigh scattering from nitrogen within the same probe volume. If the broadband emission were the wings of elastic light, which could happen if scattered light from the laser leaked into the optical assembly, e.g., by incidence on the interference filter at an oblique angle, the laser beam should be weakly visible in the direct imaging results shown in Fig. 6. However, besides as a barely visible tip in the center of the notch in Fig. 2a, this is not the case with the laser vertically polarized, largely due to the small aperture employed. In fact, no background subtraction is necessary if the excitation laser is horizontally polarized, i.e., normal to the imaging plane. Moreover, the observed broadband emission intensity of the particles or agglomerates is not affected by laser polarization direction.

A high intensity laser beam interacting with metal particles can result in broadband emission caused by laser-induced plasmas. If no breakdown of the gas phase occurs, this is sometimes referred to as phase-selective laser-induced breakdown spectroscopy (PS-LIBS).^19^ The broadband continuum emission (bremsstrahlung) is usually accompanied by Stark-broadened, but relatively narrow emission lines from neutral and ionized atoms in the gas-phase, depending on the plasma conditions. In our case, the only narrow spectral features present within the observed spectral region, between approximately 400 and 750 nm, is the nitrogen Raman peak at 607 nm. Depending on the plasma conditions, multiple ionization levels of the relevant elements (Au, Ag, or Cu and N) may be present. The fact that we do not observe any atomic or ionic emission lines makes PS-LIBS an unlikely explanation for the observed emission.

Laser-induced incandescence (LII), a common diagnostic technique for soot particles, has been applied to silver nanoparticles among other metals.^20^ A crucial aspect to the interpretation of LII spectra is the emissivity of the particles. Whereas the emissivity of soot is usually assumed to be spectrally flat, the emissivity of spherical plasmonic nanoparticles can be taken as the Mie absorption efficiency, meaning that the LII emission spectrum will be modulated by the plasmon resonance of the particles. The LII emission spectrum would therefore be the Planck curve for the particle surface temperature, spectrally modulated by the Mie absorption efficiency,^21^ and weighted by the particle size distribution for polydisperse particles. If both LII and photoluminescence are modulated by the plasmon resonance, which acts to couple light out of and into the nanoparticle by way of collective oscillations of the conduction electrons, as proposed by Dulkeith et al. regarding photoluminescence,^7^ photoluminescence and LII could appear spectrally similar. Using pulsed laser excitation, LII and photoluminescence can potentially be distinguished based on emission lifetimes. While conducting LII on gas-borne gold and silver nanoparticles, Talebi-Moghaddam et al. recently studied the broadband emission from nanoparticles excited with a pulsed (ns) laser operating at 1064 nm.^22^ The emission was attributed to multiphoton-induced upconversion photoluminescence rather than LII, due to its instantaneous response and power-law dependence on laser fluence. Typical peak intensities for pulsed lasers used in LII (10 ns pulse duration, 1 mJ pulse energy) are in the order of 10^6^ to 10^8 ^W cm^−2^,^20^ or approximately two to four orders of magnitude stronger than where we start to observe the broadband signal.

Gold and silver nanostructures are frequently used as active substrates in SERS. While SERS spectra of molecules adsorbed on gold surfaces typically exhibit relatively narrow features, they are commonly accompanied by a broad background that has been attributed to photoluminescence of the substrate, spectrally modulated by the surface plasmon resonance.^9,23^ In a clean nitrogen environment, using pure metal particles generated in a spark discharge, we do not anticipate any foreign species that could exhibit a detectable Raman signal besides nitrogen.

From the above discussion, we conclude that the observed broadband emission from gold nanoparticles is more likely caused by photoluminescence. This can be further supported on the basis of the relatively weaker signals associated with copper and silver, and the equivalent power-dependent data shown in Fig. 7 for gold. Further experiments with better control of size and shape of the particles would be valuable.

It is noteworthy that we observe photoluminescence emission on the short wavelength side of the excitation wavelength. Mooradian attributed the high-energy part of the photoluminescence spectrum from bulk gold to thermal smearing.^8^ Neupane et al. observed photon upconversion photoluminescence in gold nanoparticles when excited at the longer wavelength side of the plasmon resonance.^24^ Hugall and Baumberg observed broadband emission on both sides of the 785 nm excitation laser wavelength in relation to SERS on gold nanostructures.^25^ It was proposed that the anti-Stokes emission was caused by inelastic scattering by electrons and was shown to increase in intensity with temperature.

As already mentioned, the particles coming out of the aerosol generator are generally not individual spherical particles but agglomerates. Due to laser-induced melting, it is conceivable that some particles obtain a spherical shape while traversing the laser beam. Moreover, it is likely that the optical cross-section of the particles is reduced because of coalescence and recrystallization of the agglomerates into spherical nanocrystals and altered further by full melting. Bulk gold has a melting temperature of 1337 K, but the melting temperature is generally lower for small particles.^26,27^ Pulsed laser (fs) melting of gold nanorods was reported to result in reshaping into spherical particles at temperatures as low as 250 ℃.^28^ From energy conservation considerations, and assuming spherical particles, we can estimate the steady-state temperature of spherical particles at 15 W excitation power to approximately 500 K for 40 nm diameter particles, or 1400 K for 80 nm particles. From this simple analysis, most of the particles on the right wing of the particle size distribution shown in Fig. 3 are likely melted by the laser. Furthermore, these particles can be expected to account for the majority of the observed signal.

Finally, it should be noted that both our imaging and spectrally resolved results were collected under relatively small solid angle, and increasing the collection solid angle would result in a correspondingly increased signal intensity.

## Conclusion

We observed far-field broadband inelastic emission from gold, copper, and silver nanoparticles in aerosol phase carried in a nitrogen gas flow when excited using a weakly focused 532 nm CW laser. We attribute the laser-induced emission to plasmon enhanced photoluminescence from the nanoparticles. The photoluminescence is characterized as a broad distribution extending over the entire visible wavelength range covering both the higher and lower energy side of the excitation wavelength. We attribute the emission on the high-energy side of the excitation wavelength to thermal effects. Gold was used to demonstrate an experimental approach to detecting and potentially characterizing nanoparticles in the gas phase. The spectral shape of the photoluminescence emission is independent of excitation power. Furthermore, the intensity of the emission is independent of laser polarization, unlike spontaneous Raman and elastic Mie and Rayleigh scattering which are highly polarized. The intensity of the broadband emission is comparable to the nitrogen Raman signal. Using the nitrogen Raman as intensity reference, we have determined the quantum yield of the photoluminescence from gold nanoparticles. Although the broadband emission is attributed to plasmon enhanced photoluminescence, the exact origin and the governing mechanism is still to be further clarified, e.g., using temporally resolved measurements using a pulsed excitation laser.

An uncertainty is introduced through the less well-known particle shape and size distribution used in the calculation of the quantum yield. An improvement to the accuracy can be expected if fully coalesced and size-selected nanoparticles were used for optical measurements.

We envision these results useful for quantitative in situ optical diagnostics in aerosol-based metal nanoparticle generation and metal particle seeded semiconductor nanoparticle and nanowire growth environments. Photoluminescence may furthermore be a useful tool for studying optical and photothermal properties of single gold nanoparticles.^29,30^
